# 22 years of Minimal Incision Vertical Endoscopic Lift: a journey in endoscopic facial rejuvenation

**DOI:** 10.3389/fsurg.2025.1634862

**Published:** 2025-09-22

**Authors:** Alessandro Gennai, Mattia Colli, Leonardo Gaggio

**Affiliations:** ^1^ Private Practice “Studio Gennai”, Aesthetic and Reconstructive Plastic Surgery, Bologna, Italy; ^2^ Private Practice “Podgora7”, Milan, Italy; ^3^ Facial Plastic Surgery, University of Parma, Parma, Italy

**Keywords:** endoscopic facelift, eye lift, eyebrow lift, facial rejuvenation, aging, retinaculum, lateral canthus

## Abstract

**Introduction:**

The Minimal Incision Vertical Endoscopic Lifting (MIVEL) is a minimally invasive facial rejuvenation technique that repositions tissues with minimal scarring, reduced recovery time, and long-lasting results, while achieving a natural appearance. This study aims to describe the updated MIVEL procedure, focusing on its evolution over 22 years and providing methodological details. This includes refining the indications, identifying key fixation points for reproducible results, and highlighting the long-term success of MIVEL as an effective approach for facial rejuvenation with minimal scarring and reduced complications.

**Methods:**

This retrospective analysis encompasses a comprehensive review of 784 patients who underwent MIVEL between 2001 and 2023. The MIVEL technique involves small incisions, endoscopic guidance, and vertical lifting to reposition facial tissues. Preoperative and postoperative photographs, patients' demographic data, and complication rates were analyzed. Included patients were those seeking facial rejuvenation without extensive skin excision and attending all follow-up visits up to at least one year postoperatively. Patients were categorized into three MIVEL groups (I, II, III) of dissection, based on their age and related aging signs.

**Results:**

The average age of patients was 50.5 years, with the majority falling in the MIVEL II group (53.1 years). MIVEL I was primarily performed on younger patients (21–35 years), while MIVEL III was reserved for those over 55 years. Adjunctive procedures, such as guided Superficial Enhanced Fat Fluid Injection (SEFFI), blepharoplasty, and neck lift, were commonly combined with the MIVEL procedure. The complication rates were low, with transient issues like neuropraxia and periocular ecchymosis being the most common. No cases of severe complications like skin necrosis or permanent nerve injury were reported.

**Discussion:**

MIVEL has proven to be a highly effective and well-tolerated technique for the rejuvenation of the upper and middle thirds of the face. Its minimally invasive nature reduces recovery times and minimal scarring while providing lasting and natural-looking results. The 22-year experience underscores the reliability and high patient satisfaction associated with MIVEL, making it a preferred choice for facial rejuvenation surgery. Future directions include further refining the technique and exploring its applicability to other facial and neck rejuvenation areas.

## 1 Introduction

Facial rejuvenation surgery has evolved significantly over the last few decades, with advancements in techniques that minimize invasiveness while delivering natural and lasting results (1). Traditional facelift procedures, though effective, often come with the risk of visible scarring, longer recovery times, and complications such as unnatural aesthetic outcomes (2). These drawbacks have spurred the development of less invasive methods that aim to provide a more harmonious and youthful appearance with minimal disruption to the skin's natural anatomy (3–6).

The MIVEL (Minimal Incisions Vertical Endoscopic Lift) is an advanced, minimally invasive surgical procedure for facial rejuvenation. The endoscopically assisted minimal scalp incisions enable a comprehensive periocular lift, addressing age-related tissue descent, ptosis, and volume loss in the periocular region. Thus, by restoring the brow, temporal region, and midface in harmony, MIVEL achieves subtle yet significant rejuvenation with reduced scarring (7).

This endoscopic technique combines the redistribution and restoration of facial volumes and the repositioning of soft tissues naturally with moderate skin tension. This approach is less invasive, reduces skin tension, and minimizes (or even eliminates) the need for skin excision. The procedure's unique vertical lifting strategy addresses sagging tissues and provides long-lasting results, improving the patient's aesthetic outcome and overall satisfaction (8).

Over time, it has become objectively evident that precise fixation points are necessary to achieve an upward and backward lift of the midface, particularly for patients who desire an elongated and elevated lateral canthus with concomitant lifting of the eyebrow and temporal region (9). This demand has led us to expand the original indications of MIVEL to include the management of the lateral canthal, lower eyelid, and malar regions, and to define precise fixation areas to ensure reproducible and lasting aesthetic results (7, 8).

This article aims to describe these safe fixation areas, and provide a detailed methodological guide for the MIVEL procedure. The objective is also to underscore the long-term success of MIVEL as an established method for facial rejuvenation, offering lasting results with a low incidence of complications.

## 2 Materials and methods

This study is a retrospective assessment of data collected from 2001 to 2023. A detailed history was taken, and basic examinations were performed to ensure all patients had good overall health.

Included patients were those seeking facial rejuvenation without extensive skin excision and attending all follow-up visits up to at least one year postoperatively. The exclusion criteria included unrealistic expectations regarding the aesthetic outcome of the procedure, pregnancy or breastfeeding, medical conditions impairing wound healing, active fever, severe pulmonary diseases, active cutaneous or systemic infections, prior exposure to radiation or chemotherapy, and alcohol or drug abuse within six months before surgery.

Technical exclusion criteria included the absence of hair, as this would make endoscopic access incisions visible.

The authors followed the ethical principles laid down in the Declaration of Helsinki.

**Figure 1:**
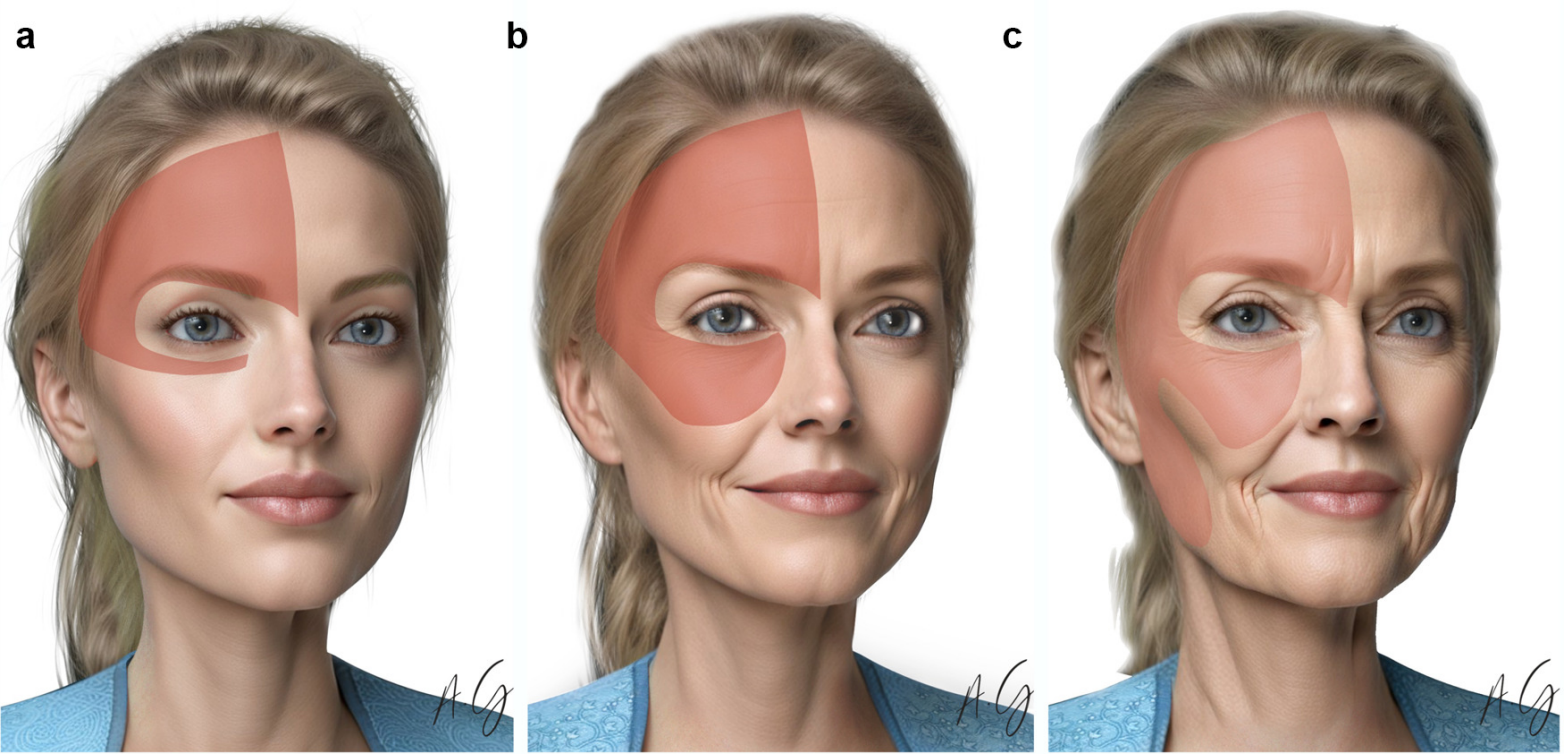
MIVEL types. **(a)** MIVEL I, **(b)** MIVEL II, **(c)** MIVEL III prototype candidate patients with the endoscopic dissection area highlighted in red.

Appropriate candidates for the MIVEL procedure were divided into three groups based on clinical examination, including physical examination of skin excess and assessment of facial areas with volume loss, to determine the treatment required to achieve optimal facial rejuvenation (Figure 1).

The study was conducted in accordance with the Declaration of Helsinki. All patients provided written informed consent prior to participation, including consent for the use and publication of clinical images. Ethical review and approval were waived for this study as it is a retrospective analysis of a surgical procedure utilizing anonymized patient data collected from existing medical records in a private practice setting. No prospective data collection was performed. Given the study's observational nature and the absence of control groups, it does not pose additional risks to patient privacy or well-being. All data were handled in compliance with applicable data protection regulations.

### 2.1 MIVEL indications

The MIVEL can be categorized into three levels of surgical dissection.

#### 2.1.1 MIVEL I

The MIVEL I technique is primarily employed when correction is focused on the periocular region and is typically indicated for young to middle-aged patients, generally between 20 and 35 years of age. Ideal candidates often present with low eyebrow positioning—whether at the tail, body, or head—along with a rounded eye shape that may be congenital or the result of previous surgical interventions. Additional common features include wrinkles at the nasal root caused by hypertonicity of the procerus and corrugator muscles, deep horizontal forehead lines, periocular wrinkles, lateral canthal ptosis, and moderate scleral show.

This patient cohort undergoes a combination of endoscopic brow rotation, external canthal repositioning and microfat grafting through guided Superficial Enhanced Fat Fluid Injection (SEFFI) using the SEFFILLER® medical device (10; 18).

The average operative time ranges from 1.5 to 2 h.

#### 2.1.2 MIVEL II

The MIVEL II procedure is typically performed in middle-aged patients, between 40 and 55 years of age, when the indications for MIVEL I are present alongside additional age-related changes. These include an increased distance between the lower eyelid and the cheek, a noticeable descent of the malar region, and a deepening of the nasolabial fold. Candidates for this technique generally exhibit only minimal laxity of the neck skin, as reported in previous studies (7, 10). The procedure usually lasts between 2 and 3 h.

The MIVEL II procedures can be combined with a limited posterior auricular and a pre-tragal incision for a jawline-neck lift. The primary surgical objectives include achieving a well-defined jawline through SMAS plication and, if necessary, applying a neck artificial ligament (NAL) to effectively address visible platysmal bands (11). When the excess neck skin was redraped to the mastoid area, skin bunching occurred at the peri-lobular area. This bunching of skin was removed along a pretragal incision. There was no incision in the sideburns or extension into the temporal hairline (7, 8, 10).

#### 2.1.3 MIVEL III

This procedure was typically performed in middle-aged or elderly patients who presented with the conditions mentioned for MIVEL I and MIVEL II, along with moderate jawline laxity. MIVEL III was indicated when there was enough laxity of the lower face associated with neck skin requiring resection. The typical patient age for this cohort was over 55 years, presenting with jowls and a moderate to large amount of neck skin laxity.

In MIVEL III procedures, the endoscopic dissection extends below the zygomatic arch in a plane above the superficial musculoaponeurotic system (SMAS), reaching the jawline.

This technique involves a deep dissection within the safety corridor anterior to the tragus and posterior to Pitanguy's line on the zygomatic arch. The dissection continues inferiorly as subcutaneous to avoid traversing the parotid fascia, extending to the mandibular angle. An endoscopic approach is then employed to lift the platysma-SMAS flap, which is anchored to the deep temporal fascia behind the temporal incision using multiple absorbable suture (2-3/0 Vycril®). This procedure typically requires between 5 and 8 h to complete.

### 2.2 Surgical planning and preoperative markings

#### 2.2.1 Division of the forehead

With the patient standing and gravity pulling down tissues, anatomical structures, soft tissue positioning, and symmetry can be accurately assessed (12).

The initial step involves dividing the forehead into two halves by drawing a vertical midline. This is a central reference point throughout the procedure, ensuring alignment and proportionality (7, 8, 10).

#### 2.2.2 Identification and marking of key anatomical landmarks

Accurate identification of key anatomical landmarks is essential to achieve both functional and aesthetic outcomes during the procedure. The temporal fusion crest, a palpable ridge between the temporalis muscle and the frontal bone, is marked to define the lateral boundaries of the forehead. The zygomatic arch is also identified and marked to serve as a reference for both the trajectory of the facial nerve and the aesthetic contour of the midface. Careful marking of the superior and inferior orbital rims helps delineate the orbital framework, ensuring precision during dissection. The supraorbital notch is palpated to locate the exit point of the supraorbital nerve, which is then marked to reduce the risk of nerve injury. Similarly, identification and marking of the supratrochlear notch allow for the protection of the supratrochlear nerve throughout the procedure.

#### 2.2.3 Marking the incision sites

Strategic marking of the incision sites is crucial to ensure optimal surgical access while minimizing visible scarring. A central incision measuring 1.5 cm is marked just behind the hairline and serves as the primary access point for the procedure. In addition, two paramedian incisions of the same length are placed approximately 4–5 cm lateral to the central incision to provide symmetrical access. To facilitate lateral dissection without compromising aesthetic results, two temporal incisions, each 3 cm in length, are marked inferior to the temporal fusion crest and positioned 1.5 cm behind the hairline.

#### 2.2.4 Drawing the Pitanguy line

The Pitanguy line is a crucial guide for identifying the course of the facial nerve (cranial nerve VII) as it traverses the zygomatic arch. This line is drawn from the earlobe to approximately 1.5 cm above the eyebrow's tail (13). By marking this trajectory, the surgeon can avoid inadvertent nerve damage, preserving both motor function and facial symmetry (7, 10).

**Figure 2:**
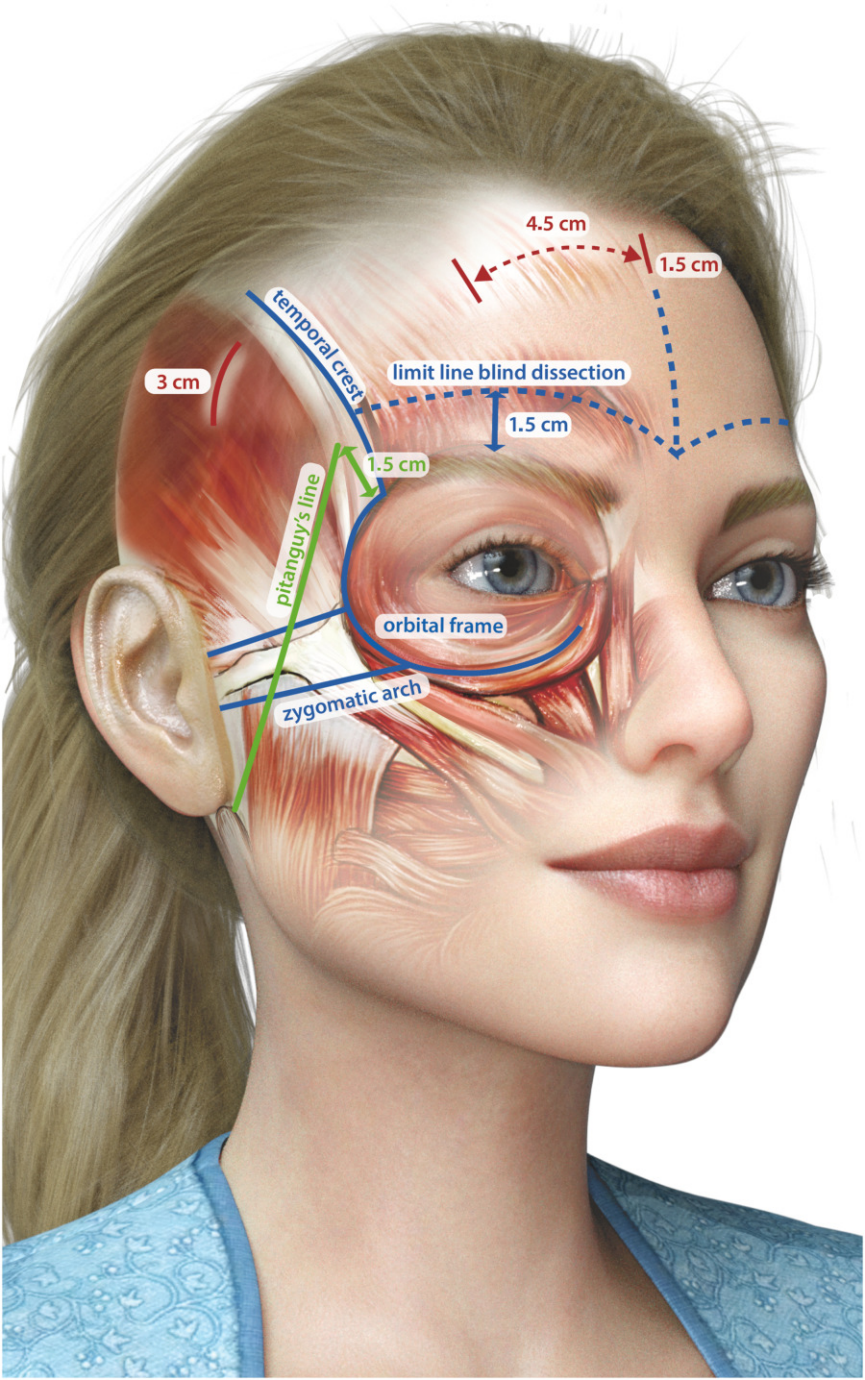
Preoperative markings.

A visual representation of preoperative markings is presented in Figure 2.

### 2.3 Anesthesia and infiltration

The MIVEL procedure can be performed under general anesthesia or monitored intravenous sedation. During the onset of sedation, the hair is braided and secured to a sterile drape using sutures following the incision planning (7, 10).

The surgeon administers tumescent local anesthesia to the dissection areas. Typically, a saline solution with lidocaine and adrenaline (1:400,000) is used for the frontal, temporal, and malar regions. For the upper periorbital area, lateral canthus, glabella, zygomatic arch, and incision sites, a 1:100,000 dilution is used. In areas such as the forehead, the volume of local anesthesia is carefully limited to minimize swelling. The procedure begins with subcutaneous injections and infiltration along the dissection plane at the temple incisions. Subsequently, the paramedian incisions are infiltrated subcutaneously and deeply at the periosteal level. The anesthetic solution is then administered along the temporal crest fusion line in the subperiosteal plane, extending inferiorly towards the brow. Deep periosteal injections follow along the orbital rims and glabellar region. Finally, the superior and inferior zygomatic arches receive subcutaneous injections to complete the anesthesia protocol (7, 10).

### 2.4 Surgical details and technical considerations

Operating following a precise sequence of events is critical to the overall outcome of the operation. The aim is to maximize the vertical shift of tissue before the lateral tightening of the SMAS/platysma and skin excision in the neck, when required. This is essential for precision and to enhance each maneuver's effectiveness and synergistic effects. Procedural steps are described here in the sequence in which they should be performed.

#### 2.4.1 Blind dissection of the sub-periosteal plane of the forehead and on top of the deep temporalis fascia

**Figure 3:**
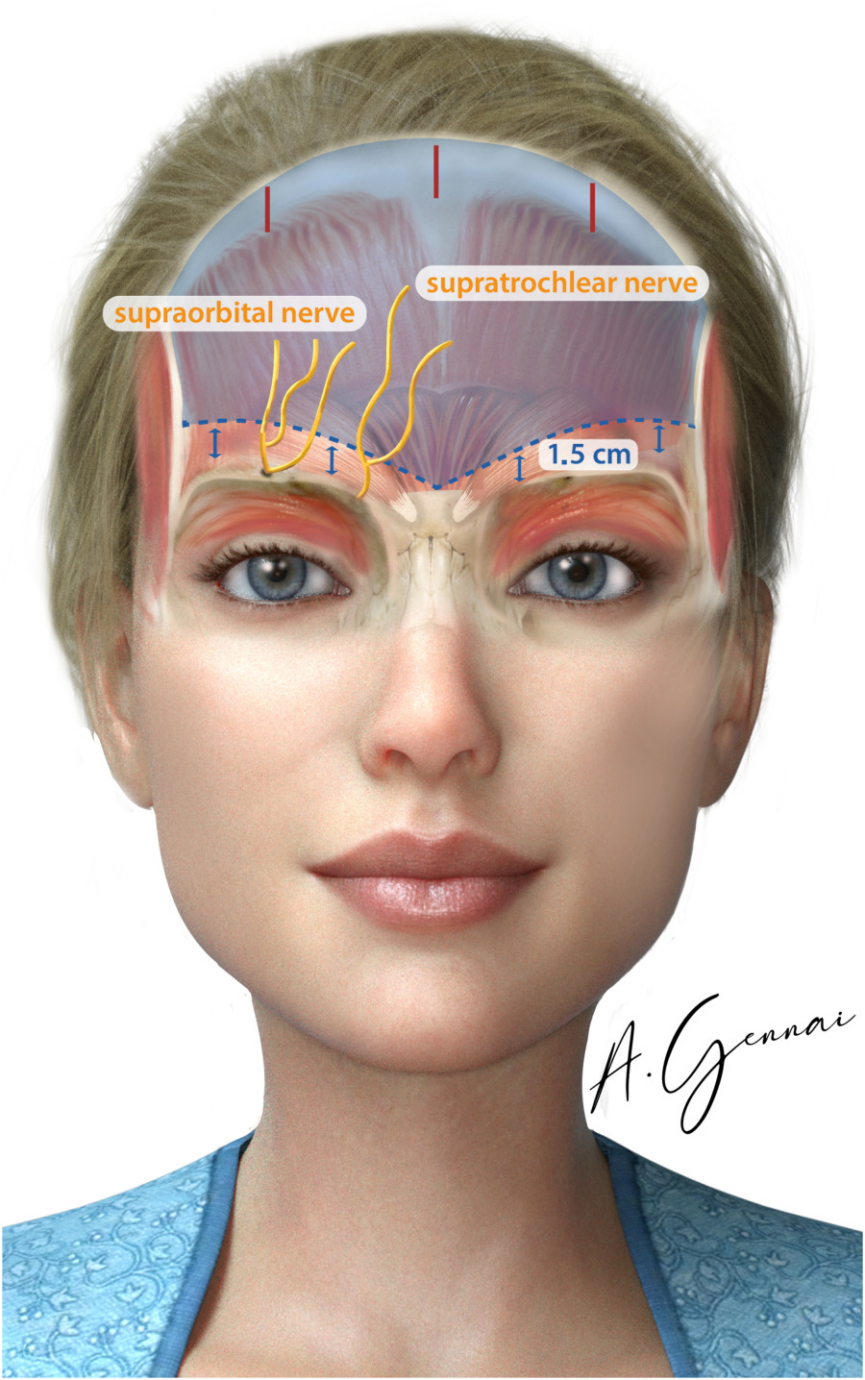
Blind sub-periosteal dissection of the forehead marked in blue.

The initial phase of the dissection is carried out without the aid of an endoscope. The surgeon operates on the subperiosteal plane in the forehead region and above the temporalis fascia in the temporal area (Figure 3). On the forehead, the dissection is carefully performed while maintaining a minimum distance of 1.5 cm above the orbital rim to avoid compromising critical structures. In the temporal region, the surgical plane remains confined within the temporal-auricular adhesion to ensure precise and safe tissue handling.

After completing the blind dissection and establishing a unified optical pocket in both the frontal and temporal regions, the endoscope is carefully introduced to ensure precision and safety throughout the procedure.

#### 2.4.2 Temporal endoscopic dissection

The endoscopic dissection begins in the temporal area, where it is crucial to completely elevate the superficial temporal fascia. This step provides access to the plane above the deep temporal fascia, a critical protective layer safeguarding the frontal branch of the facial nerve (14). Maintaining this specific plane is essential, as it minimizes the complete release of the temporal crest adhesion to connect the temporal and forehead dissection areas.

As the blunt dissection progresses toward the lateral corner of the eyelid, the sentinel vein emerges as a vital anatomical landmark. This vein should be meticulously preserved or cautiously coagulated to avoid unnecessary trauma. The zygomatic-facial neurovascular bundle is lateral to the sentinel vein, encompassing both nerve and vein structures. Further laterally are the zygomatic-temporal vein and nerve. Between the sentinel vein and the zygomatic-facial bundle is the pathway to access to the suborbicularis oculi fat (SOOF), a significant fat pad that provides volume and contour to the midface (15). Meanwhile, the pathway to the midface itself is situated between the zygomatic-facial and zygomatic-temporal bundles. Medial to the sentinel vein are the lateral retinaculum and the lateral canthal tendon, key structures for eyelid and canthal position.

#### 2.4.3 Periocular dissection

Once the dissection progresses across the zygomatic arch, the SOOF is carefully elevated, and the lateral retinaculum is released. This step facilitates the safe elevation of the lower eyelid tissues, the lateral canthus, and the lateral cheek.

#### 2.4.4 Advanced dissection (MIVEL III)

**Figure 4:**
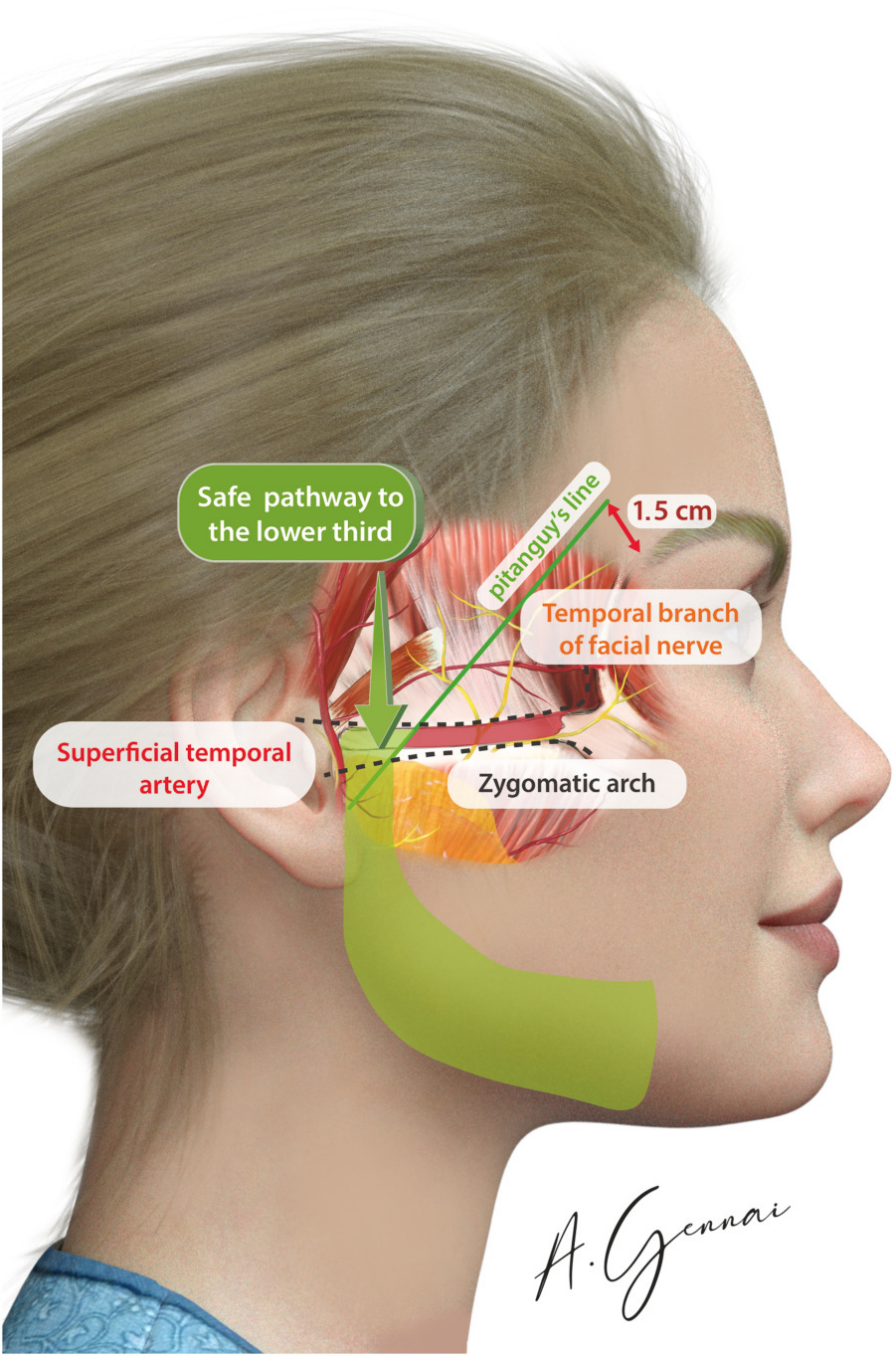
Safe pathway to the lower third in the MIVEL III procedure marked in green.

In MIVEL III procedures, the endoscopic dissection extends below the zygomatic arch, proceeding in a plane above the SMAS and advancing inferiorly toward the jawline (Figure 4). This approach allows for effective mobilization and repositioning of the facial soft tissues while minimizing trauma to underlying structures. A key maneuver in this technique involves precise dissection above the zygomatic arch within a subcutaneous plane. The dissection proceeds posterior to the Pitanguy line and anterior to the tragus, carefully navigating this well-defined anatomical corridor. This pathway ensures the preservation of vital facial nerve branches and associated vascular structures, reducing the risk of functional or aesthetic complications.

#### 2.4.5 Forehead endoscopic dissection

In the subperiosteal plane, special care is needed to maintain the integrity of the periosteum while advancing toward the orbital rim. Using a blunt dissection technique, the periosteum is elevated systematically from lateral to medial, ensuring the supraorbital neurovascular bundle is exposed. Periosteal strands are meticulously released on all sides, including posterior to the nerve, to fully mobilize the forehead flap without tension or restriction.

For the glabella and muscle dissection, the approach focuses on releasing and elevating the corrugator supercilii and procerus muscles. This step involves precise isolation of the supratrochlear nerve as it courses through the fibers of the corrugator muscle. Complete periosteal release, combined with careful preservation and isolation of the sensory nerves, ensures optimal mobility of the forehead tissue. This meticulous technique minimizes tension and allows for a controlled elevation of the forehead flap.

#### 2.4.6 Vertical suspension and fixation

The long-term success of suspensions is not primarily due to the device used but to the extensive mobilization of tissues and the subsequent healing process (16). If an anatomical structure is pulled without adequate mobilization, any device used to secure it will likely fail within a relatively short period. Conversely, if the structure is fully mobilized, the fixation should be performed with a simple absorbable suture 2/0 or 3/0 (Vycril®); the goal of the fixation is to hold the structure in its new position for the time needed for healing and the formation of fibrotic scar tissue, which ultimately ensures long-term stability.

#### 2.4.7 Jawline fixation (MIVEL III)

**Figure 5:**
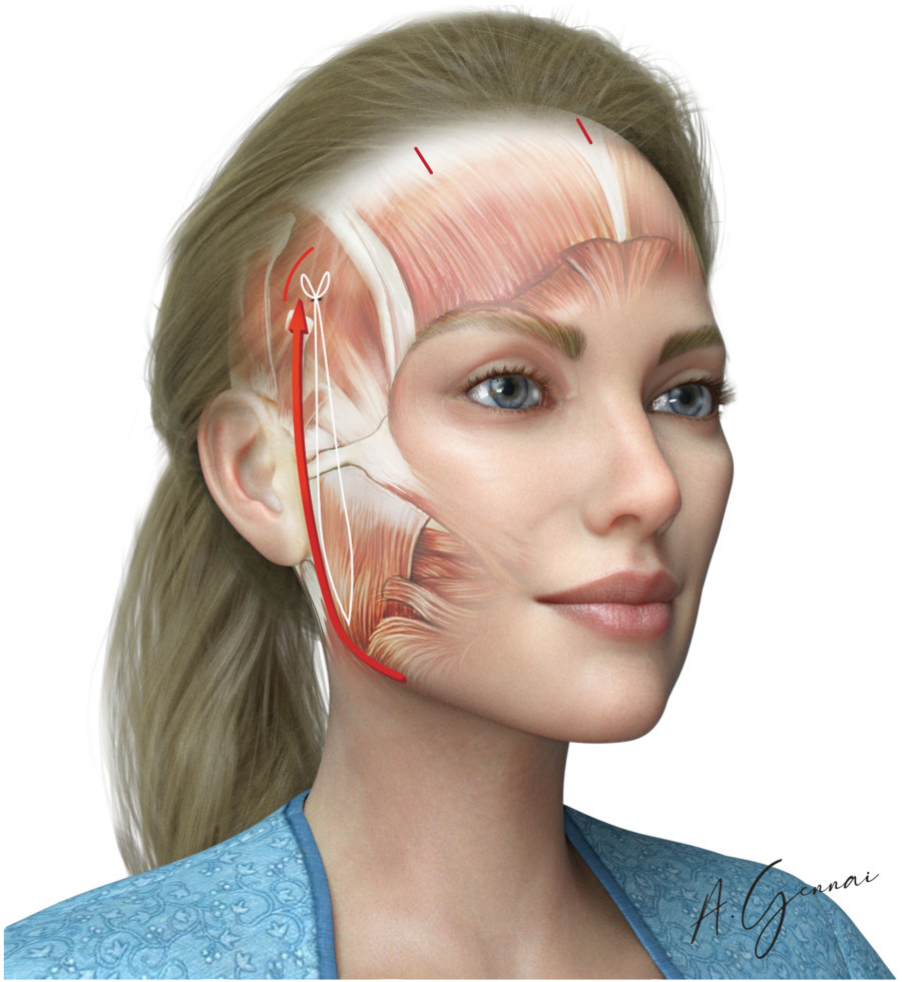
Jawline fixation in the MIVEL III procedure.

Jawline fixation is performed exclusively in MIVEL III. The primary objective of this procedure is to reposition and retighten the jawline along a vertical vector, specifically indicated in cases of mild laxity in this area. A 3 cm incision is made around the earlobe to access the SMAS overlying the mandibular angle through subcutaneous dissection: this access facilitates the positioning of the stitch. The ptotic tissues of the jawline are then lifted using 2/0 absorbable sutures (Vicryl® 2/0), securing the selected point. The suture is passed through the pretragal dissection, extending above the previously described zygomatic arch (Figure 5). Finally, after verifying the tissue lifting vector and its corresponding effect, the suture is anchored to a point on the deep temporal fascia.

#### 2.4.8 Malar fixation

**Figure 6:**
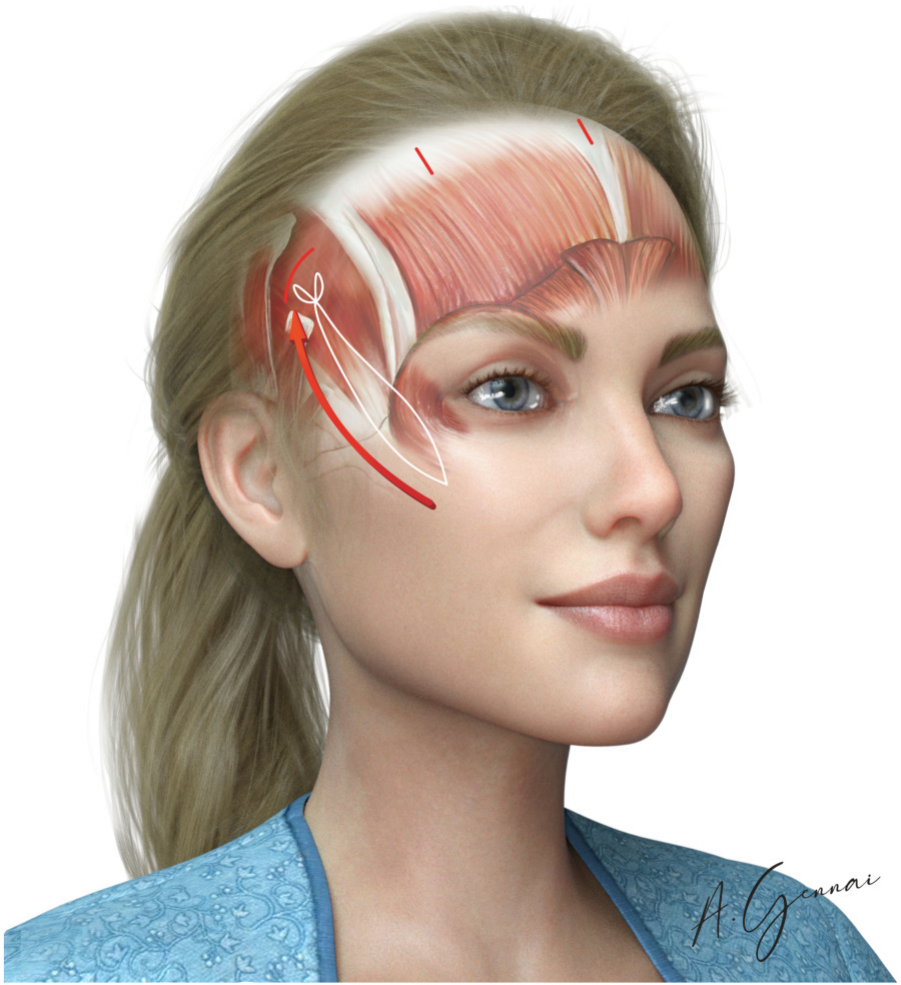
Malar fixation.

The primary aim of the malar fixation is to reposition the cheek region along a vertical vector (Figure 6). This suspension is performed in MIVEL II and III only in cases of actual downward displacement, which must be differentiated from a mere loss of tone due to malar fat pad hollowing. In the latter case, the treatment should focus solely on restoring volume. It is important to emphasize that lifting the cheek region must be paired with a corresponding lift of the temporo-frontal portion. This is because a harmonious result in malar and zygomatic lifting cannot be achieved without repositioning the temporo-frontal zone. Techniques that aim exclusively at lifting the malar portion without addressing the temporo-frontal area are likely to reduce the openness of the gaze, resulting in an unnatural appearance (10).

Malar fixation was performed with the Gennai's stitch with absorbable 3/0 or 2/0 suture (Vycril®) suture.

#### 2.4.9 Temporal fixation (paracanthal temporal fascia point)

**Figure 7:**
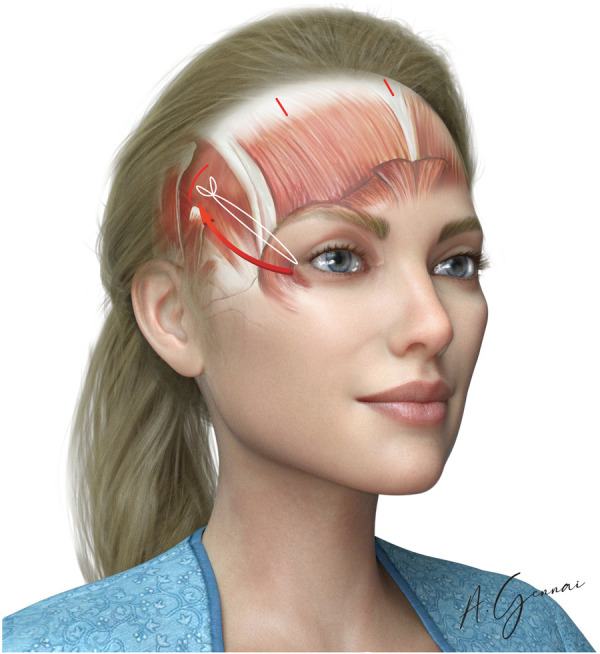
Temporal fixation.

This step is indicated when aiming to achieve an “eye-lengthening effect”. After a complete dissection and release of the external canthal ligament, the suture is applied at this level using the endoscope for visualization. The suture secures the upper part of the resected canthal ligament and is anchored to the proper fascia of the frontalis muscle (Figure 7). Para canthal fixation was performed with the absorbable 3/0 suture (Vycril®).

#### 2.4.10 Deep temporal fixation

**Figure 8:**
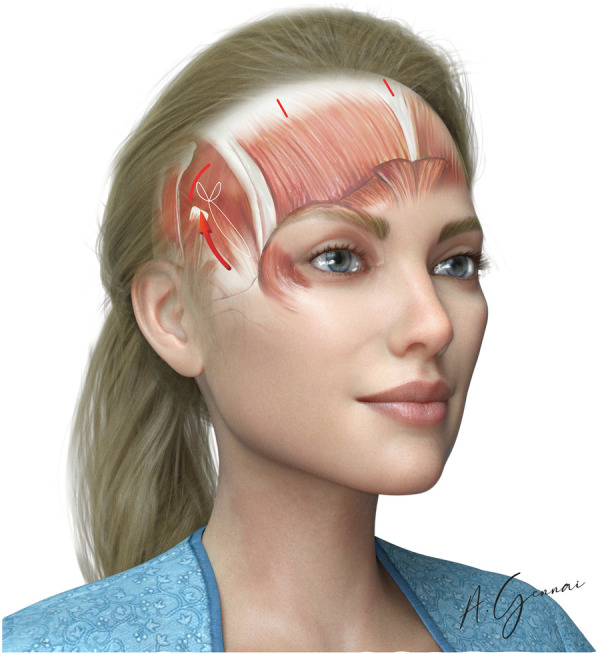
Deep temporal fixation.

Temporal fixation of the deep subcutaneous tissues of the elevated flap to the deep temporal fascia is performed with an absorbable 2/0 or 3/0 suture (Vicryl®). This fixation elevates the tail of the brow, the temporo-zygomatic area, and the corner of the eyelids, elongating the eyelid fissure (Figure 8).

#### 2.4.11 Bicoronal fixation

**Figure 9:**
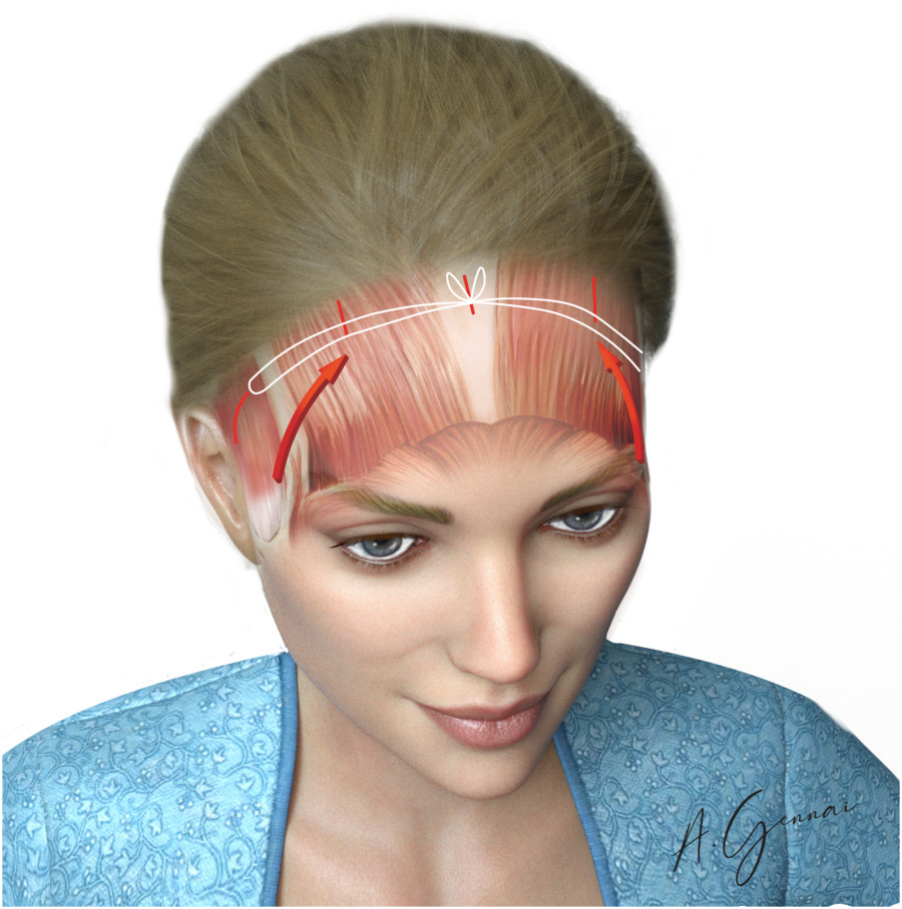
Bicoronal fixation.

Bicoronal fixation is used to enhance the brow's tail elevation effect. It is performed with a non-absorbable suture (0 Mersilene®) passed through the medial angle of the temporal incisions and tied through the midline incision (Figure 9).

#### 2.4.12 Paramedian suspension and fixation

The primary objective of this step is to achieve a durable lift of the lateral third of the eyebrow. However, the long-term success of this suspension is significantly compromised if there is an incomplete release of the orbicularis oculi muscle, subperiosteally in its frontal portion and supraperiosteally in its temporal portion. Equally important is the thorough release of the adhesions at the level of the temporal ridge, which, if left intact, can impose persistent tension and limit the extent of mobilization.

Achieving this complete release requires direct visualization, as blind dissection in these areas carries a substantial risk of injury to critical neurovascular structures, particularly the trochlear and supraorbital nerves.

The Gennai's stitch technique is effective for paramedian fixation. This approach begins with two small horizontal stab wound incisions made at the same level on the forehead, one on each side, positioned below the paramedian incisions. Following, a Reverdin needle is passed from the forehead incision under the scalp, emerging at the paramedian incision. Once the needle exits at the paramedian incision, it is loaded with suture material and brought back through the initial forehead incision. The tip of the Reverdin needle is then carefully used to grasp the subcutaneous tissue and elevated periosteum below the skin at the forehead incision. The suture needle is passed below the scalp and exits again at the paramedian incision, freed from the Reverdin needle. Subsequently, the suture needle grasps the deep tissue at the posterior edge of the paramedian incision. The two incisions are approximated by tightening the suture, creating a temporary fold of redundant skin between the forehead and scalp incisions.

**Figure 10:**
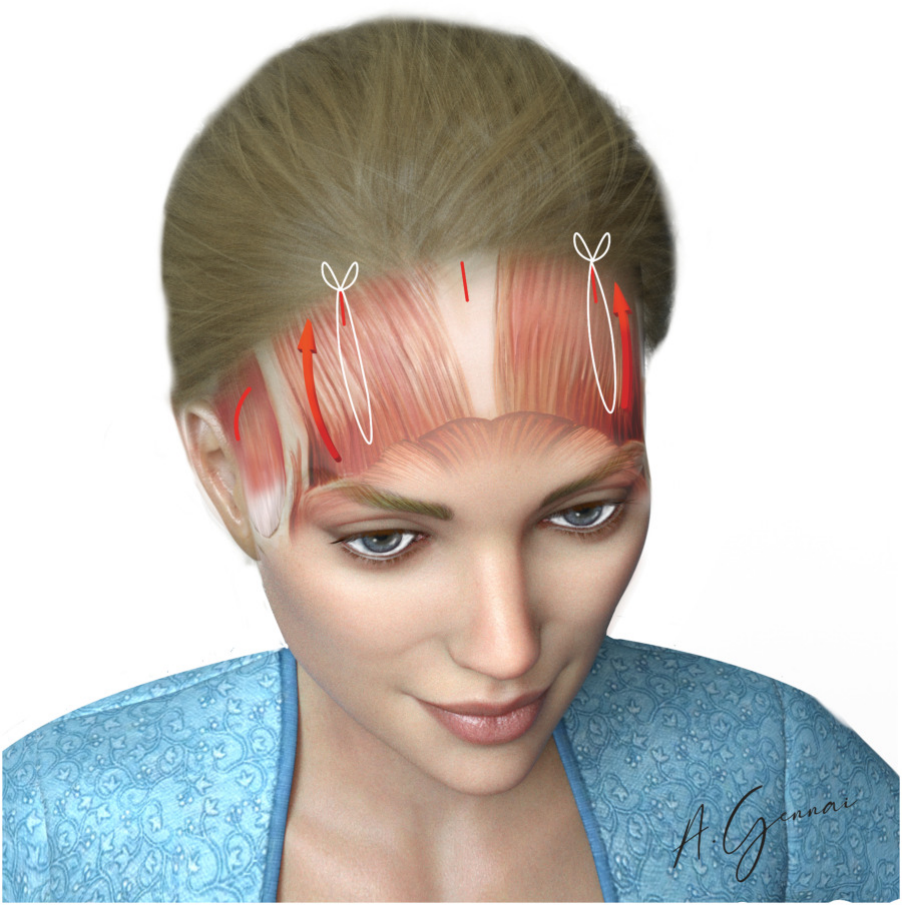
Paramedian fixation.

Once a tension-free flap has been elevated, paramedian fixation allows the tissue to be securely held in its new position while enabling precise control over the final brow shape (Figure 10). The position of the fixation determines the distribution of tension across the brow. For instance, placing the fixation more lateral to the midline emphasizes the elevation of the brow tail, whereas positioning it closer to the midline exerts a more pronounced effect on the brow body.

The paramedian fixation point is typically positioned approximately 5 cm from the midline in female patients and 4 cm in males. Lower entry points on the forehead generally result in stronger traction and more noticeable skin folds, particularly in patients with heavier or more prominent foreheads.

The Gennai's stitch, when executed with precision using absorbable 2/0 or 3/0 suture (Vycril®) mounted on a Reverdin needle, provides robust and durable fixation. This method ensures a harmonious brow contour with a natural arch and elevated tail, effectively achieving functional and aesthetic goals.

#### 2.4.13 Skin closure and postoperative protocol

The skin closure of the scalp is performed using 4–0 Silk sutures. Typically, a single suture is sufficient for both the central and paramedian incisions, while three sutures are employed for each temporal incision. To maintain the skin in an anti-gravitational position, Steri-Strips are applied over the malar and forehead areas. Following this, the incisions are covered with sterile gauze. To further support the surgical area and minimize postoperative edema, Reston foam is applied over the forehead and temporal regions. Finally, a mild tension bandage is positioned to secure the dressing and is maintained in place for four days. This comprehensive approach is designed to ensure effective wound healing by reducing edema in the dissected area and promoting periosteal adhesion, thereby contributing to optimal surgical outcomes.

Patients are advised to rest supine for the first 48 h to facilitate lymphatic drainage and prevent fluid accumulation, particularly given the extensive scalp undermining performed posterior to the vertex.

The first postoperative check is scheduled for the fourth day after surgery. During this visit, the initial bandage applied during the procedure is carefully removed. The surgical incisions on the scalp are then disinfected and medicated and a removable elastic forehead bandage is applied to provide support and reduce swelling. The next follow-up visit takes place approximately ten days after surgery. This visit is primarily dedicated to the removal of stitches. One week after the intervention, patients are allowed to take a warm shower and gently dry their hair. Social reintegration is usually possible two weeks after the procedure, allowing patients to feel more comfortable resuming their regular social activities.

Long-term follow-up is essential to monitor the progression of healing and assess the final outcomes of the procedure. For this purpose, patients are scheduled for subsequent visits at one month, three months, five months, and one year postoperatively. These follow-ups enabled the medical team to evaluate the success of the intervention, collect photographic documentation, address any concerns, and provide further guidance as needed.

## 3 Results

### 3.1 Patient demographics

The patients included in the study were followed for a minimum of 1 year postoperatively. The results were analyzed by means of descriptive statistics.

A total of 784 patients (737 performed by the senior surgeon AG) underwent the MIVEL procedure, classified into three groups: MIVEL I, MIVEL II, and MIVEL III (Table 1).

**Table 1:** Patient demographic characteristics and distribution by procedure.

Patient characteristics (*n* = 784)	*n* (%)	Average age, years (range)
Total	784 (100%)	50.5 (21–72)
Sex		
Female	752 (95.9%)	50.3 (21–72)
Male	32 (4.1%)	55.7 (48–64)
MIVEL I	88 (11.2%)	28.0 (21–35)
MIVEL II	632 (80.6%)	54.1 (33–72)
+ SMAS plication mandibular mini-lift	221 (35.0% of MIVEL II)	51.5 (45–60)
+ NAL cervical lift	126 (19.9% of MIVEL II)	63.5 (55–72)
MIVEL III	64 (8.2%)	48.5 (40–57)

NAL, Neck Artificial Ligament; SMAS, Superficial Musculoaponeurotic System.

The average age in each group reflects the link between patient age and the selection of specific surgical techniques (Table 1).

The MIVEL I group included 88 patients with a mean age of 28 years (Table 1). This cohort primarily consisted of younger individuals seeking early intervention for facial ptosis or aesthetic refinement of the upper third of the face.

The MIVEL II group was the largest, with 632 patients and a mean age of 54.1 years. This group represented a more diverse patient population, including individuals seeking more advanced facial rejuvenation, often with moderate to severe soft tissue descent of the upper and middle third of the face. The shift toward later ages in this subgroup suggests that more advanced surgical interventions are typically reserved for patients exhibiting more pronounced aging signs.

The MIVEL III group comprised 64 patients, with a mean age of 48.5 years (Table 1). Patients in this category typically sought correction for midface and lower facial sagging, often as an alternative to more invasive procedures. We began performing the MIVEL III surgical procedure in 2022, hence the low number of patients.

### 3.2 Associated surgical procedures combined with MIVEL

The MIVEL technique is frequently performed in combination with other surgical procedures to enhance overall facial rejuvenation and optimize patient outcomes (Table 1). Patients undergoing MIVEL I frequently received additional procedures to address the upper face and periorbital region. The most commonly associated procedure was SEFFI, performed in 75% of cases, providing volume restoration and skin quality improvement. Additionally, transconjunctival lower blepharoplasty was performed in 39.8% of patients to address lower eyelid fat herniation while preserving the integrity of the orbital septum.

Given that MIVEL II is primarily indicated for patients with more advanced signs of aging in the upper and midface, various complementary procedures were integrated. In this cohort, we combined the procedure with a SMAS-platysma plication mandibular mini-lift to improve lower face contouring in 35% of cases or a NAL cervical lift technique to address neck laxity in 20% of cases. The selection of mini-lift within this age group suggests that less invasive techniques are preferred in the earlier stages of facial aging. Upper blepharoplasty was performed in 15% of cases to correct upper eyelid dermatochalasis, while transconjunctival lower blepharoplasty was indicated in 60% of patients for fat repositioning. Mini-pinch blepharoplasty was performed in 44.9% of cases for minor skin redundancy correction. SEFFI was applied in 80.1% of patients to restore lost volume and to regenerate tissues through stem cells derived from autologous adipose tissue.

A higher proportion of complementary procedures were adopted for patients undergoing MIVEL III, primarily targeting midface and lower face sagging. Upper blepharoplasty was performed in 37.5% of cases, while transconjunctival lower blepharoplasty was necessary in 65.3% of patients. Additionally, skin-only lower blepharoplasty was performed in 53.1% of cases. Guided SEFFI was the most frequently associated procedure, performed in 81.2% of patients.

### 3.3 Visual outcomes

**Figure 11:**
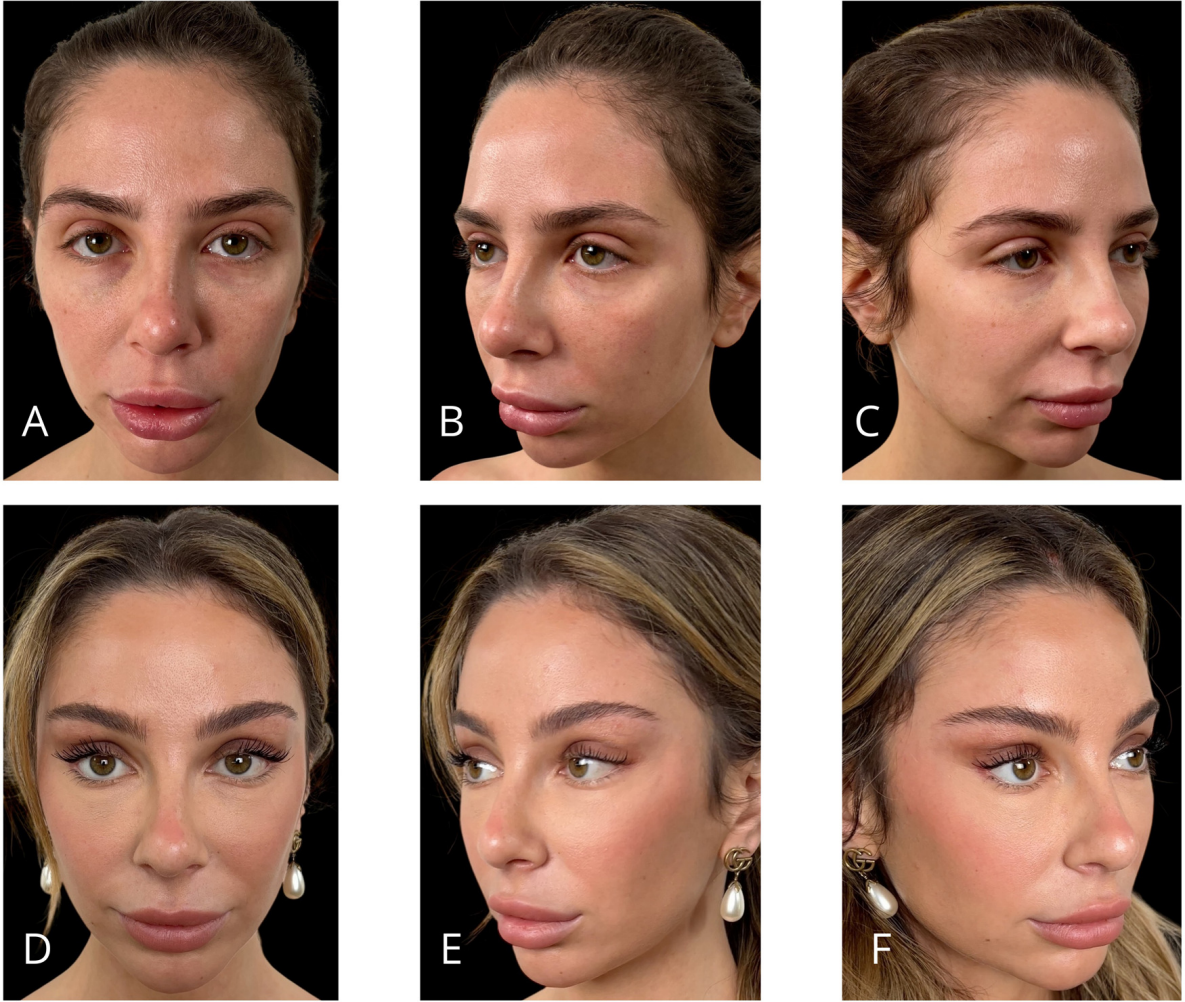
Representative result of MIVEL I technique combined with SEFFI in a 29-year-old female patient. Panels (**A–C**) show the patient preoperatively; panels (**D–F**) demonstrate the results 5 months postoperatively. Endoscopic dissection was performed in the forehead and peri-orbital frame. Fixations with 2/0 absorbable sutures included temporal fixation (paracanthal temporal fascia point) and deep temporal fixation. The bicoronal suture was performed using a non-absorbable suture. SEFFI technique was applied to the inferior eyelid sulcus, malar, and zygomatic areas: microfat injection was performed in the sub-orbicularis plane in the inferior eyelid sulcus and in the subcutaneous plane in the malar and zygomatic regions.

**Figure 12:**
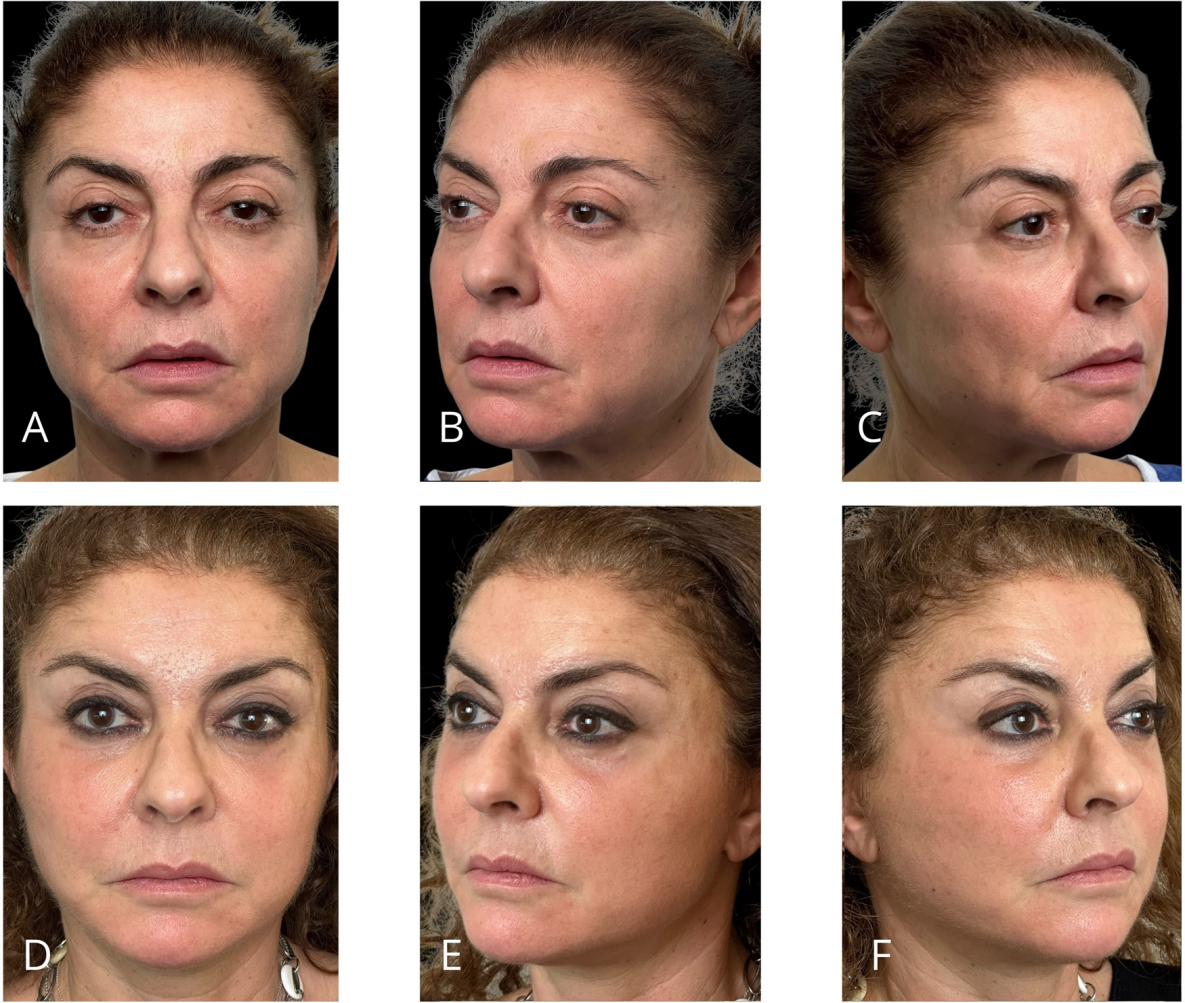
Representative result of MIVEL II technique combined with SMAS-platysma plication mini-lift, skin mini-pinch lower blepharoplasty, and SEFFI procedure in a 57-year-old female patient. Panels **(A–C)** show the patient preoperatively; panels **(D–F)** demonstrate the results 5 months postoperatively. Endoscopic dissection was performed in the forehead, peri-orbital frame, and malar and zygomatic areas. Fixations with 2/0 absorbable sutures included temporal fixation (paracanthal temporal fascia point), deep temporal fixation and malar fixation. The bicoronal suture was performed using a non-absorbable suture. The SEFFI technique was applied to the inferior eyelid sulcus, malar and zygomatic areas, and jawline: microfat was injected in the sub-orbicularis plane in the inferior eyelid sulcus, in the subcutaneous plane in the malar and zygomatic areas, and in the deep fat compartments of the malar region. A preseptal transconjunctival blepharoplasty was combined with a mini-pinch skin resection of the lower eyelids. The jawline mini-lift involved subcutaneous dissection above the SMAS, SMAS plication, skin resection, and suturing.

**Figure 13:**
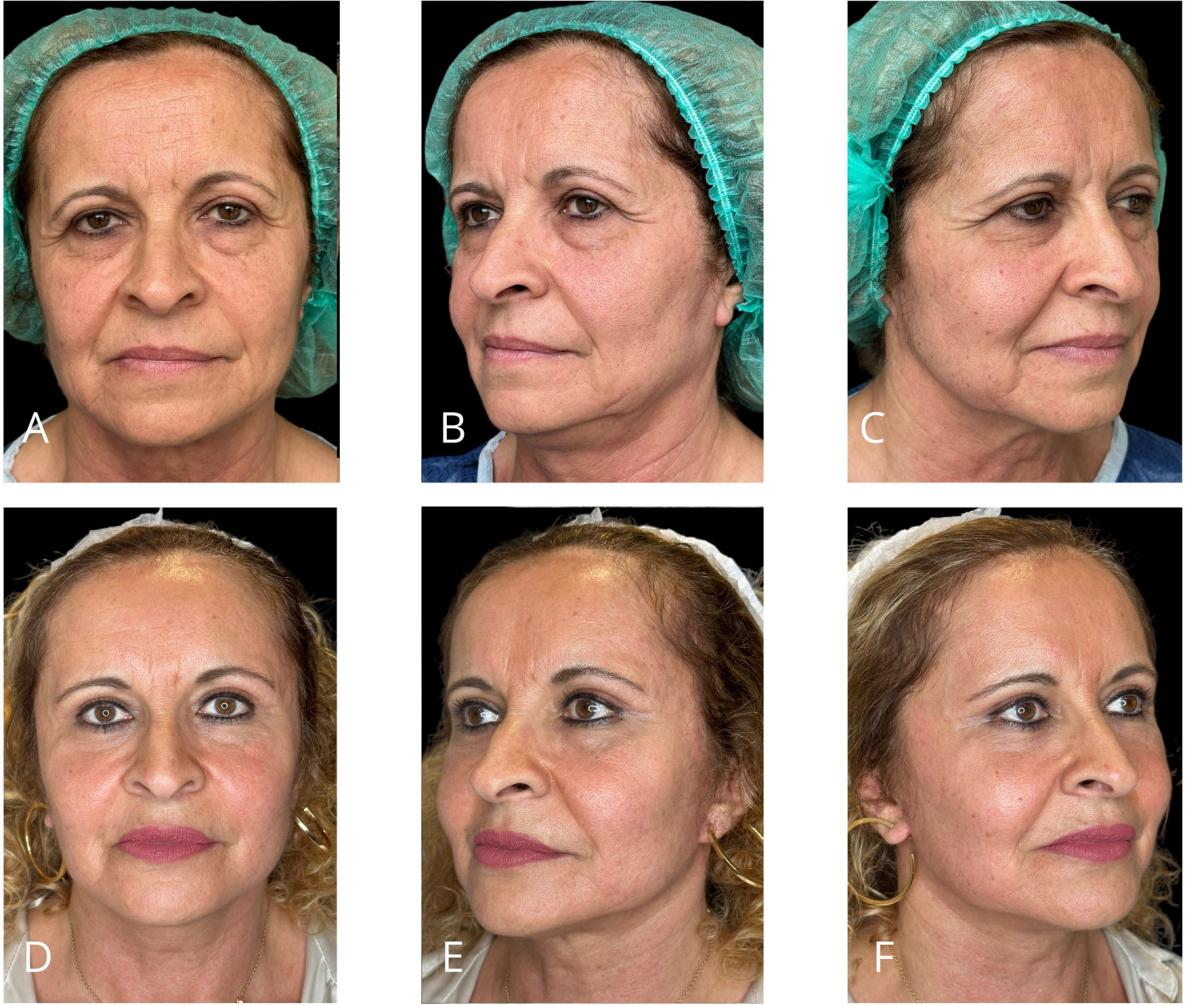
Representative result of MIVEL II technique combined with NAL neck lift, skin mini-pinch lower blepharoplasty, upper blepharoplasty, and SEFFI procedures in a 63-year-old female patient. Panels **(A–C)** show the patient preoperatively; panels **(D–F)** demonstrate the results 5 months postoperatively. Endoscopic dissection was performed in the forehead, peri-orbital frame, and malar and zygomatic areas. Fixations with 2/0 absorbable sutures included temporal fixation (paracanthal temporal fascia point), deep temporal fixation and malar fixation. The bicoronal suture was performed using a non-absorbable suture. The SEFFI technique was applied to the inferior eyelid sulcus, malar and zygomatic areas, and jawline: microfat was injected into the sub-orbicularis plane in the inferior eyelid sulcus, the subcutaneous plane in the malar and zygomatic areas, and the deep fat compartments in the malar region. The upper blepharoplasty involved only skin resection. A preseptal transconjunctival lower blepharoplasty was combined with a mini-pinch skin resection. The NAL neck lift included a wide subcutaneous dissection of the cheek, jawline, and neck above the SMAS and platysma. An artificial ligament (PTFE Gore™ patch) was sutured to the platysma in the medial region and anchored to the mastoid area (one per side). The ligaments were buried within the platysma using absorbable running sutures. Excess skin was resected and closed.

**Figure 14:**
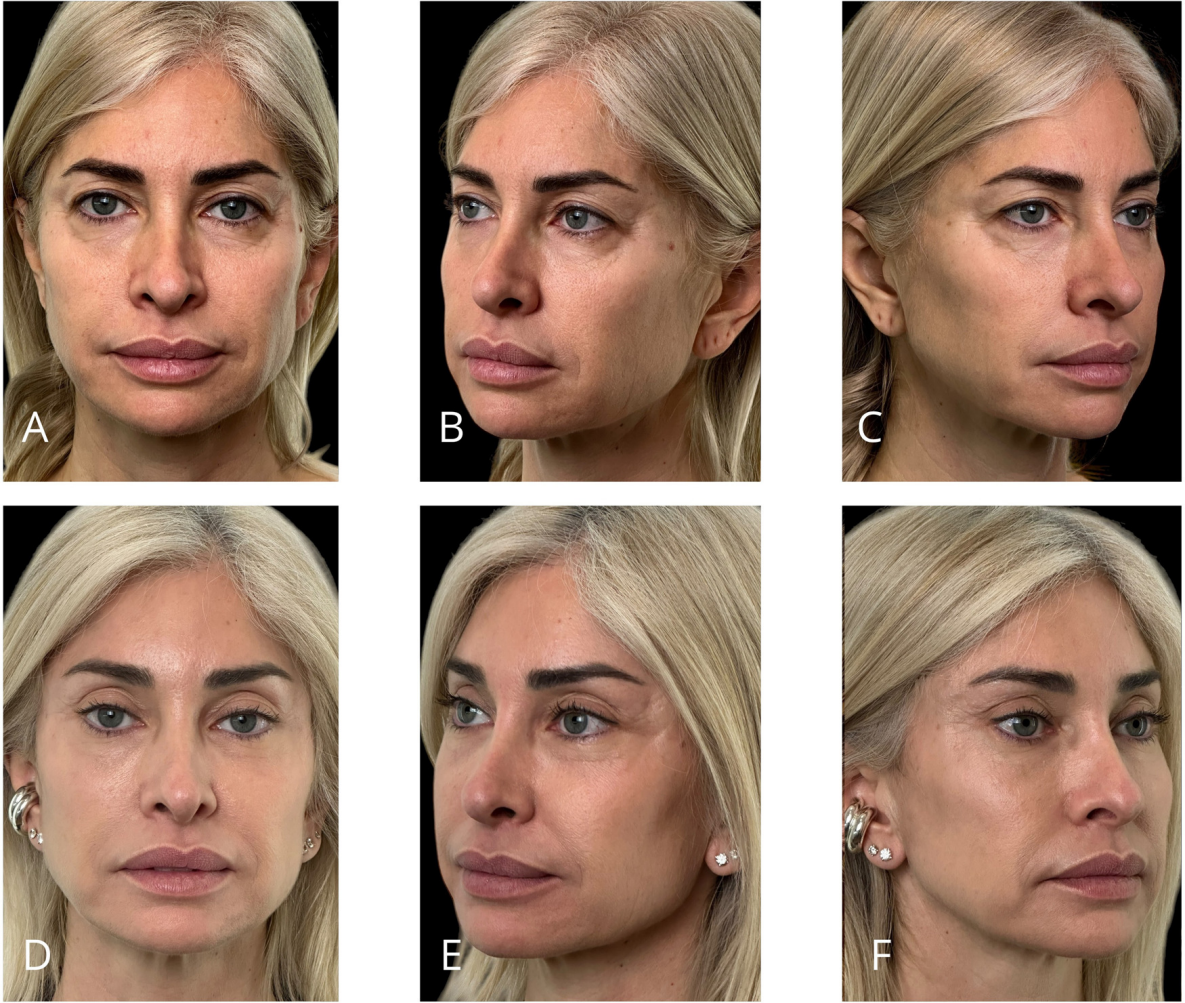
Representative result of MIVEL III technique combined with skin mini-pinch, lower blepharoplasty, and SEFFI procedures in a 45-year-old female patient. Panels (**A–C**) show the patient preoperatively; panels (**D–F**) demonstrate the results 5 months postoperatively. Endoscopic dissection was performed in the forehead, peri-orbital frame, malar and zygomatic regions, and jawline. Fixations with 2/0 absorbable sutures included temporal fixation (paracanthal temporal fascia point), deep temporal fixation, vertical fixation, and malar fixation. The bicoronal suture was performed using a non-absorbable suture. The SEFFI technique was applied to the inferior eyelid sulcus, malar and zygomatic areas, and jawline: microfat was injected into the sub-orbicularis plane in the inferior eyelid sulcus, and into the subcutaneous plane in the malar and zygomatic areas. A preseptal transconjunctival blepharoplasty was performed in combination with a mini-pinch skin resection of the lower eyelids.

Representative outcomes of the different procedures are shown in Figures 11–14.

### 3.4 Safety analysis

All perioperative and postoperative complications, including reoperations and reasons for revisions, were recorded in a prospective patient database and are summarized in Table 2. The patients included in the study were followed for a minimum of 1 year postoperatively. Our postoperative evaluation included follow-ups after one month, three months, 5 months and one year. No postoperative skin flap necrosis, hematoma, seroma, or sialocele cases were recorded. Transient neuropraxia of the frontal branch of the facial nerve developed in 7 patients and spontaneously resolved in 6–10 weeks. No permanent nerve injuries were recorded. Infection or delayed surgical wound healing occurred in 5 cases and required regular local disinfection and antibiotics. There were no cases of unsatisfied aesthetic needs requiring an additional surgical touch-up procedure.

**Table 2:** Complications and revisions.

Complication	Prevalence—*n* (%)	Revision/Intervention
Postoperative skin flap necrosis	0 (0%)	
Hematoma	0 (0%)	
Seroma	0 (0%)	
Infection/delayed healing	5 (0.6%)	Local disinfection and antibiotics
Sialocele	0 (0%)	
Transient neuropraxia of the frontal branch of the facial nerve	7 (0.9%)	None, resolved after 6–10 weeks
Transient neuropraxia of the hypoglossal nerve	0 (0%)	
Transient alopecia at the incision site	157 (20.0%)	Local minoxidil therapy, resolved after 3–6 months
Transient asymmetry of the lower lid with scleral show	0 (0%)	
Periocular ecchymosis	690 (88.0%)	
Prolonged edema (more than 15 days)	149 (19.0%)	None, resolved after 5 weeks
Skin fovea in the area of the fixation point	635 (81.0%)	None, resolved after 4 weeks
Canthal asymmetry	71 (9.1%)	None, resolved after 3–6 months
Transient paresthesia of the forehead and scalp	55 (7.0%)	None, resolved after 3–6 months

## 4 Discussion

Facial aging is more than the descent and stretch of tissues. Some areas of the face expand and contract, giving the perception that the facial structures are falling (17). The focus of modern facelifting has therefore shifted to provide necessary volume restoration and overall facial shaping. In fact, some plastic surgeons have adopted fat grafting and autologous regenerative therapy as supplements to facelift surgery (18, 19).

We believe that the most effective approach to surgical facial rejuvenation involves an antigravitational repositioning of the tissues, primarily using vertical vectors. In contrast, the traditional facelift technique, which utilizes a preauricular approach, predominantly repositions tissues with a lateral vector, resulting in minimal effect on the frontotemporal-malar region. In this regard we also consider the frontotemporal-malar and periorbital region to be a central area in achieving comprehensive facial rejuvenation. Addressing this region is essential to obtain harmonious and natural results, as it significantly contributes to the overall youthful appearance of the face.

Additionally, the classic incisions in facelift techniques can cause noticeable scarring in front of the ears, shortening of sideburns, and distortion of the ear's anatomy (10). Therefore, the signs of these traditional procedures are becoming easy to recognize, generating a stigma for the patients (20). To overcome these limitations, the MACS (minimal-access cranial suspension), a vertical vector–based facelift concept of pull was developed, representing a significant improvement over the traditional SMAS facelift.

However, while looping sutures achieve a vertical suspension of the malar region, they do not provide a vertical repositioning of the frontotemporal-malar region (21).

The MADE (minimal-access deep-plane extended) lift approach was then developed, combining MACS and the deep plane technique (22). In this procedure, the incisions are placed near the sideburns and tragal area, demanding a high level of precision, and possibly generating easily recognizable scars.

Instead in our endoscopic facelift concept, achieving vertical repositioning of the fronto-temporal-malar and periorbital region requires complete and thorough release of the periosteum in the frontal portion and the orbicularis muscle in the lateral-inferior orbital frame. Such a complete dissection can be performed effectively and safely, while preserving vascular and nerve structures, only through endoscopic-assisted dissection.

In this regard a comparison can be drawn with the technique described by Kao et al., who presented the Ponytail Lift based on over 600 cases of endoscopic deep-plane facial rejuvenation. Their approach involves extensive sub-SMAS dissection, comprehensive release of retaining ligaments, and vertical repositioning of facial soft tissues through four hidden scalp incisions, aiming for global vertical lifting with minimal visible scarring (23).

Both the Ponytail Lift and MIVEL techniques involve, in addition to a subperiosteal forehead dissection, a midface dissection within the sub-SMAS plane: superiorly, between the suborbicularis oculi fat and the orbicularis oculi muscle, and inferiorly, along the zygomaticus major and minor muscles, extending across the nasolabial fold. However, unlike the broad and complete deep-plane release performed in the Ponytail Lift, the MIVEL technique emphasizes selective ligament release while preserving key anchoring structures, such as the zygomatic and masseteric ligaments, to maintain natural contour and avoid overcorrection.

One of the key distinctions lies in the suspension and fixation system. Kao's method employs two non-absorbable bicoronal sutures and a multiple cable sutures system anchored securely to the deep temporal fascia. In the Ponytail Lift, sculpting islands of superficial temporal fascia is also a mandatory step to enable effective vertical traction transmission (23). In contrast, MIVEL employs a single bicoronal non-absorbable suture but uses absorbable sutures for facial tissues suspension anchored to the deep temporal fascia. These sutures are placed through minimal stab incisions, and the vector and fixation points are individually customized. Optional forehead paramedian fixation may be used to elevate the central brow, while lateral fixation addresses brow tail ptosis. In MIVEL III, additional suspension of the platysma by means of a cable suspension suture is incorporated as part of the extended rejuvenation protocol.

Regarding neck treatment, the Ponytail Lift incorporates both supraplatysmal and subplatysmal dissection, facilitating anterior platysma plication as well as a posterior platysmal corset, where the lateral edge of the platysma is anchored to the posterior neck fascia (23). The MIVEL technique, by contrast, requires only a supraplatysmal dissection, followed by optional platysma plication. In cases presenting with increased bulk of the subplatysmal tissues or with significant platysmal banding, the use of a neck artificial ligament (NAL) is considered to enhance cervical contour and structural support (11).

The MIVEL technique prioritizes natural and harmonious results by focusing on the anatomical repositioning of deeper structures rather than merely tightening the skin, thus minimizing the risk of unnatural, overstretched appearances commonly associated with traditional facelifts. In addition, we believe that the long-term stability of the results does not derive from the quality or strength of the suspension sutures but rather from the scar adhesion of tissues in their new position, maintained by absorbable fixation sutures.

In our technique, skin resection is not required, as the lifting effect is not achieved through skin traction but through tissue repositioning. A minimal skin resection is performed only when executing a SMAS-platysma plication lift or NAL neck lift. In these cases, the repositioning of the SMAS and platysma may lead to mild skin redundancy at the pre-tragal and retro-auricular levels. Thus, according to our experience, extending the pretragal incision into the temporal region is not necessary to achieve adequate skin resection. The upper two-thirds of the face suffers from deflation and descent in aging, leading to the need for resuspension and redraping of tissue rather than excision.

Moreover, the newly developed MIVEL III allows for lifting and tightening of the jowls and jawline, resulting in a heart-shaped, tapered face, while maintaining all the advantages of the classical MIVEL without scar in the pre-tragal region. Continued development and adaptation of MIVEL will focus on enhancing outcomes and expanding its applications to other areas of facial and neck rejuvenation.

By performing an extended endoscopic dissection, the MIVEL technique achieves superior results in the midface, where many short-scar techniques fail, while preserving the natural blood supply to the skin and facial tissues. Additionally, MIVEL can be safely performed alongside other procedures for the delivery of shape and support, such as microfat transfer through guided SEFFI.

The results from this 22-year retrospective study of 784 patients undergoing MIVEL reveal a clear progression in the choice of surgical interventions as age increases. MIVEL I is predominantly performed on younger patients (21–35 years, average 27), while middle-aged patients choose MIVEL III (48–64 years, average 56). MIVEL II, the broadest category, accommodates a wide age spectrum but is primarily selected by middle-aged patients (33–72 years, average 53).

⁠The structured progression of surgical choice based on age confirms that our aesthetic procedures are tailored to the degree of facial aging, with less invasive approaches preferred at earlier stages and more comprehensive procedures reserved for older patients.

Our analysis demonstrates that MIVEL procedures have globally low complications. Specifically, hematoma, seroma, skin necrosis, and tuck-up rates are below the numbers reported in the literature (24).

Furthermore, our cohort presented a distinctly lower tuck-up rate for surgical correction of less than 2%, compared to around 50% with SMASectomy, more than 20% for SMAS plication, and around 4% for the MADE lift (24–26). A possible explanation is that the skin tends to get thinned out in traditional facelift procedures, leading to atrophy and a loss of support over time. In contrast to MIVEL, which preserves the natural blood supply to the tissues, traditional facelift procedures may compromise vascular integrity. This can lead to subcutaneous tissue shrinkage and laxity of the overlying skin.

Endoscopic facelift approaches report a frontal branch neuropraxia rate of about 4% in expert hands (27) while we report in our study cohort a rate of less of 1% of transient neuropraxias, resolved within 6–12 weeks.

Although the results from this study are promising, there are several limitations that must be considered. One limitation is the retrospective nature of the analysis, which may introduce biases in patient selection and data collection. The lack of objective measures of patient-reported outcomes, such as quality of life or long-term satisfaction, is another limitation. Future studies incorporating these measures would further validate the effectiveness of MIVEL.

However, the findings from this extensive study confirm the reliability and effectiveness of the MIVEL technique in achieving stable, long-lasting results in facial rejuvenation. The repositioned facial structures maintain their improved aesthetic appearance for years following the procedure, with no significant tissue descent or sagging recurrence. The strategic selection of fixation points, combined with comprehensive endoscopic mobilization, plays a crucial role in ensuring the durability of outcomes.

Overall, the combination of minimal invasiveness, high patient satisfaction and low complication rates strongly supports the superiority of MIVEL over conventional facelift techniques. These results reinforce the technique's role in achieving natural, long-lasting facial rejuvenation with minimal risks and downtime, meeting the increasing demand for natural and harmonious results.

## Data Availability

The raw data supporting the conclusions of this article will be made available by the authors, without undue reservation.
